# Endobronchial Ultrasonography With Guide Sheath for the Diagnosis of Peripheral Pulmonary Lesions in Japan: A Literature Review

**DOI:** 10.7759/cureus.55595

**Published:** 2024-03-05

**Authors:** Daisuke Minami, Nagio Takigawa, Daisuke Himeji

**Affiliations:** 1 Respiratory Medicine, Hosoya Hospital, Okayama, JPN; 2 Internal Medicine, Kawasaki Medical School, Okayama, JPN; 3 General Internal Medicine 4, Kawasaki Medical School, Okayama, JPN; 4 Internal Medicine, Miyazaki Prefectural Miyazaki Hospital, Miyazaki, JPN

**Keywords:** diagnosis identification, guide sheath, peripheral pulmonary lesions, endobronchial ultrasonography, flexible bronchoscopy

## Abstract

We evaluated the usefulness of endobronchial ultrasonography with guide sheath (EBUS-GS) for the diagnosis of peripheral pulmonary lesions (PPLs) in Japan. We searched the PubMed/Medline database using the keywords “EBUS guide sheath” for Japanese studies on EBUS-GS published between January 2004 and August 2023. We included 32 original articles that evaluated the diagnostic yield of EBUS-GS for PPLs. Case reports and conference abstracts were excluded due to limited information available for quality assessment. The diagnostic yield of EBUS-GS was 73.6% for 2996 malignant lesions, 65.4% for 752 ground-glass nodules, 59.4% for 414 benign lesions, 61.3% for 1114 lesions of size ≤2 cm, and 75.6% for 1246 lesions of size >2 cm; it was 69.4% for lesions located in the upper lobe (n=793), 71.9% for the middle lobe/lingula (n=121), and 62.5% for the lower lobe (n=334). None of the patients experienced severe complications. In this review, EBUS-GS is effective for the diagnosis of malignant and benign PPLs. A multimodality approach is needed to further enhance its diagnostic performance.

## Introduction and background

Recently, the increased use of imaging has led to a higher frequency of incidentally identified peripheral pulmonary lesions (PPLs). Bronchoscopy is commonly performed in Japan for the diagnosis of PPLs. In 2004, Kurimoto et al. first reported the usefulness of endobronchial ultrasonography with guide-sheath (EBUS-GS) [1]. Since then, EBUS-GS has been recognized as one of the most effective bronchoscopic methods for collecting samples from PPLs [2]. However, its diagnostic yield has varied widely among previous studies. Moreover, EBUS is increasingly used to guide sampling tools, often in combination with a guide sheath, in multiple countries outside Japan. Roth et al. reported that EBUS did not increase the detection rate of cancer in PPLs [3]. In Japan, the procedure is typically performed with a guide sheath to detect PPLs. Here, we reviewed previous studies on the usefulness of EBUS-GS for detecting PPLs conducted in Japan.

## Review

**Figure 1:**
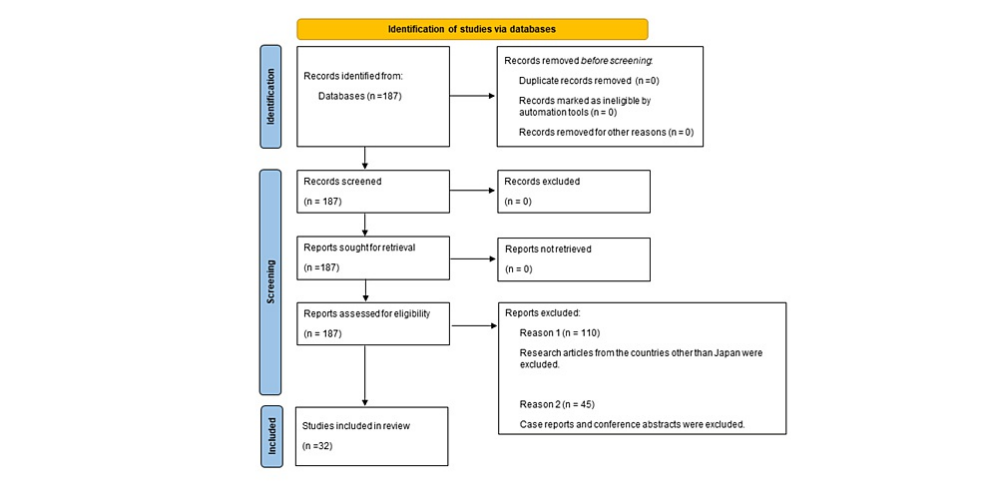
Flow diagram showing a summary of the literature search.

We systematically searched the Medline database via PubMed for studies on EBUS-GS during bronchoscopy published between January 2004 and August 2023. The search was performed using the keywords “EBUS guide sheath” and it retrieved 187 studies. After screening, we selected 77 original articles from Japan that reported the diagnostic yield of EBUS-GS for diagnosing PPLs. Case reports and conference abstracts were excluded due to limited information available for quality assessment. Ultimately, 32 studies were selected for inclusion (Figure 1) [1,4-34]. We calculated the diagnostic yield as the ratio of the number of successful diagnoses to the total number of malignant lesions. In addition, we recorded the number of ground-glass nodules, lesion size (≤2 or >2 cm), malignancy potential (benign or malignant), and lobar location. Given that we only reviewed previously published data, ethical approval was not required. Tables 1-5 present the diagnostic yields of EBUS-GS for malignant lesions, ground-glass nodules, benign lesions, malignant potential, and size. The overall yields were 73.6%, 65.4%, 59.4%, 73.2%, 61.3%, and 75.6% (for lesions of size ≤2 cm and those >2 cm), respectively. The analysis is performed using the 10 studies [1,5,8,10-11,14-15,18,24,33] for lobar location. The values were 69.4%, 71.9%, and 62.5% for those in the upper lobe, middle lobe/lingula, and lower lobe, respectively. No patients reported any severe complications.

**Table 1:** Diagnostic yields based on malignant lesions using EBUS-GS. The data from 17 studies (2996 lesions) are used for the calculation of overall diagnostic yield. On malignant lesions including lung cancer, the diagnostic yield is 73.6% (range: 61.9%-88.2%). *^1^PPL is less than 15 mm diameter. ^*2^Histopathologic diagnostic yield by ultrathin bronchoscopy. ^*3^PPL is less than 30 mm diameter. *^4^Detection rate of re-biopsy for malignant cells. *^5^Virtual bronchoscopic navigation was used for 48.7% of this study. *^6^PPLs without interstitial lung disease. EBUS-GS, endobronchial ultrasound-guided transbronchial biopsy with guide sheath; PTX, pneumothorax; PNA, pneumonia; ND, not described; BW, bronchial washing; PPLs, peripheral pulmonary lesions

No.	Study	Year design	Additional guidance	Number of lesions	Number of diagnosis	Yield (%)	Complications	Sampling method
1	Kurimoto et al [1]	2004	Fluoro	101	82	81.1	2 Moderate bleeding	Forceps, brush
2	Kikuchi et al [4]	2004	Virtual fluoro	18	12	66.7	1 PTX	Forceps, brush
3	Tamiya et al [5]	2011	Virtual fluoro	63*^1^	39	61.9	ND	Forceps, brush
4	Oki et al [6]	2012	Fluoro	82	58	70.7	2 PNA	Forceps, brush, BW
5	Minami et al [7]	2015	Virtual fluoro	60	50	83.3	2 PTX 2 PNA	Forceps, brush, BW
6	Minezawa et al [8]	2015	Fluoro	110	86	78.2	5 PTX 4 PNA 1 delirium	Forceps, brush
7	Hayama et al [9]	2015	Virtual fluoro	965	744	77.1	8 PTX 5 pulmonary infection	Forceps, brush
8	Oki et al [10]	2015	Virtual fluoro	123	100	81.3*^2^	3 PTX 1 PNA 1 chest pain	Forceps, brush, BW
9	Chavez et al [11]	2015	Fluoro	213	143	67.5	No severe complications	Forceps, brush
10	Okachi et al [12]	2016	Virtual fluoro	175*^3^	112	64.0	ND	Forceps, brush, BW
11	Izumo et al [13]	2016	Virtual fluoro	44	33*^4^	75.0	No severe complications	Forceps, brush
12	Uchimura et al [14]	2016	Virtual*^5^ fluoro	76	54	71.1	ND	Forceps, brush
13	Asano et al [15]	2017	Virtual	56	43	76.8	1 hyperventilation	Forceps, brush
14	Tachihara et al [16]	2017	Virtual fluoro	17	15	88.2	No severe complications	Forceps, brush
15	Kajikawa et al [17]	2019	Fluoro	187	137	73.3	ND	Forceps, brush, BW
16	Ito et al [18]	2022	Virtual fluoro	339*^6^	252	74.3	2 PTX 2 PNA	Forceps, brush
17	Kurihara et al [19]	2022	Fluoro	367	244	66.5	8 fever 5 hemorrhage 4 PTX, 3 PNA	Forceps, brush, BW

**Table 2:** Diagnostic yields based on ground-glass nodule using EBUS-GS. The data from six studies (752 lesions) are used for the calculation of overall diagnostic yield. The diagnostic yield is 65.4% (range: 56.7%-77.1%). ^*1^Virtual bronchoscopic navigation was used for 77.6% in this study. EBUS-GS, endobronchial ultrasound-guided transbronchial biopsy with guide sheath; PTX, pneumothorax; PNA, pneumonia; ND, not described; BW, bronchial washing

No.	Study	Year design	Additional guidance	Number of lesions	Number of diagnosis	Yield (%)	Complications	Sampling method
1	Izumo et al [20]	2013	Virtual fluoro	40	26	65.0	1 PTX	Forceps, brush, BW
2	Ikezawa et al [21]	2014	Fluoro	67	37	56.7	ND	Forceps, brush
3	Izumo et al [22]	2015	Virtual fluoro	187	116	62.0	ND	Forceps, brush
4	Nakai et al [23]	2017	Virtual fluoro	35	27	77.1	1 disinhibition	Forceps, brush
5	Nakai et al [24]	2017	Virtual*^1^ fluoro	254	167	65.7	1 disinhibition	Forceps, brush
6	Ikezawa et al [25]	2017	Virtual fluoro	169	119	68.6	2 PTX	Forceps, brush

**Table 3:** Diagnostic yields based on benign lesions using EBUS-GS. The analysis is performed using the 10 studies with a total of 414 benign lesions. The diagnostic yield is 59.4% (range: 25.0%-91.3%). ^*1^PPL is less than 15 mm diameter. ^*2^Definitive diagnosis was obtained by bronchoscopy alone or clinical features. ^*3^Histopathologic diagnostic yield by ultrathin bronchoscopy. ^*4^Contribution to clinical decision-making for hematological diseases. EBUS-GS, endobronchial ultrasound-guided transbronchial biopsy with guide sheath; PTX, pneumothorax; PNA, pneumonia; ND, no date; BW, bronchial washing; PPLs, peripheral pulmonary lesions

No.	Study	Year design	Additional guidance	Number of lesions	Number of diagnosis	Yield (%)	Complications	Sampling method
1	Kurimoto et al [1]	2004	Fluoro	49	34	69.3	2 Moderate bleeding	Forceps, brush
2	Kikuchi et al [4]	2004	Virtual fluoro	6	2	33.3	1 PTX	Forceps, brush
3	Tamiya et al [5]	2011	Virtual fuoro	52*^1^	36	69.2	ND	Forceps, brush
4	Shinagawa et al [26]	2012	Fluoro	171	99*^2^	57.8	No complications	Forceps, brush, BW
5	Oki et al [6]	2012	Fluoro	20	5	25.0	2 PNA	Forceps, brush, BW
6	Minezawa et al [8]	2015	Fluoro	39	22	56.4	5 PTX 4 PNA 1 delirium	Forceps, brush
7	Oki et al [10]	2015	Virtual fluoro	26	11	42.3*^3^	3 PTX 1 PNA 1 chest pain	Forceps, brush, BW
8	Asano et al [15]	2017	Virtual	6	5	83.3	1 Hyperventilation	Forceps, brush
9	Ito et al [18]	2022	Virtual fluoro	23*^1^	21	91.3	2 PTX 2 PNA	Forceps, brush
10	Nakashima et al [27]	2022	Virtual fluoro	22	11*^4^	50.0	No severe complications	Forceps, brush

**Table 4:** Diagnostic yields based on malignant and/or benign lesions using EBUS-GS. On malignant and/or benign lesions using EBUS-GS including 13 studies, the diagnostic yield is 73.2% (range: 61.3%-87.3%). ^*1^PPL is less than 15 mm diameter. ^*2^Histopathologic diagnostic yield by ultrathin bronchoscopy. ^*3^Virtual bronchoscopic navigation was used for 54.0% in this study. ^*4^PPL is cavitary. ^*5^PPLs in patients with interstitial lung disease. ^*6^PPLs in patients with interstitial lung disease. The lesions are distant from fibrotic lesions. ^*7^PPLs without interstitial lung disease. EBUS-GS, endobronchial ultrasound-guided transbronchial biopsy with guide sheath; PTX, pneumothorax; PNA, pneumonia; ND, no date; BW, bronchial washing; CP, chest pain; BL, bleeding; AR, arrhythmia; HY, hypoxemia; PPLs, peripheral pulmonary lesions

No.	Study	Year design	Additional guidance	Number of lesions	Number of diagnosis	Yield (%)	Complications	Sampling method
1	Kurimoto et al [1]	2004	Fluoro	150	116	77.3	1 pulmonary infection	Forceps, brush
2	Ishida et al [28]	2011	Virtual fluoro	99	80	80.8	ND	Forceps, brush
3	Tamiya et al [5]	2011	Virtual fluoro	115*^1^	75	65.2	ND	Forceps, brush
4	Ishida et al [29]	2012	Fluoro	65	42	64.6	1 PTX	Forceps, brush, BW
5	Oki et al [6]	2012	Fluoro	102	63	61.7	2 PNA	Forceps, brush, BW
6	Minezawa et al [8]	2015	Fluoro	149	108	72.5	5 PTX 4 PNA 1 delirium	Forceps, brush
7	Sakamoto et al [30]	2015	Virtual fluoro	71	62	87.3	ND	Forceps, brush
8	Oki et al [10]	2015	Virtual fluoro	150	111	74*^2^	3 PTX 1 PNA 1CP	Forceps, brush, BW
9	Hayama et al [31]	2016	Virtual*^3^ fluoro	50	40*^4^	80	2 moderate bleeding	Forceps, brush
10	Ito et al [32]	2021	Virtual fluoro	19*^5^	12	63.2	1 PTX	Forceps, brush
11	Ito et al [33]	2021	Virtual fluoro	24*^6^	20	83.3	1 PTX	Forceps, brush
12	Ito et al [18]	2022	Virtual fluoro	362*^7^	273	75.4	2 PTX 2 PNA	Forceps, brush
13	Oki et al [34]	2022	Virtual fluoro	300	203	67.7	3 PTX, 4 PNA 1 BL, 1AR, 1 HR, 1 broken GS	Forceps, brush, BW needle aspiration

**Table 5:** Diagnostic yields based on lesion size using EBUS-GS (≤2 and >2 cm). Seventeen studies reported diagnostic yields separately for lesions ≤2 and >2 cm. The diagnostic yields for 1114 lesions ≤2 cm and 1246 lesions >2 cm were 61.3% (range: 42.3%-75.9%) and 75.6% (range: 58.3%-91.7%), respectively ^*1^PPL is less than 15 mm diameter. ^*2^PPL is diagnosed with lung cancer. ^*3^Histopathologic diagnostic yield by ultrathin bronchoscopy. ^*4^PPL is less than 30 mm diameter. ^*5^PPL is ground-glass nodule. ^*6^PPLs in patients with interstitial lung disease. ^*7^PPLs in patients without interstitial lung disease. ^*8^Histopathologic diagnostic yield by guide sheath method EBUS-GS, endobronchial ultrasound-guided transbronchial biopsy with guide sheath; ND, no date; PPL, peripheral pulmonary lesion

No.	Study	Year design	Number of lesions (≤2 cm)	Number of diagnosis	Yield (%)	Number of lesions (>2 cm)	Number of diagnosis	Yield (%)
1	Kurimoto et al [1]	2004	81	59	72.8	69	57	82.6
2	Ishida et al [28]	2011	58	44	75.9	41	36	87.8
3	Tamiya et al [5]	2011	115*^1^	75	65.2	ND	ND	ND
4	Ishida et al [29]	2012	26	11	42.3	39	31	79.5
5	Oki et al [6]	2012	23*^2^	15	65.2	59*^2^	43	72.8
6	Chavez et al [11]	2015	84	52	61.9	128	91	71.0
7	Minezawa et al [8]	2015	80	51	63.8	69	57	82.6
8	Minami et al [7]	2015	23*^2^	15	65.2	37*^2^	31	83.7
9	Oki et al [10]	2015	80	52*^3^	65.0	70	59*^7^	84.2
10	Okachi et al [12]	2016	81*^2^	45	55.5	94*^2,4^	67	71.2
11	Uchimura et al [14]	2016	24*^2^	12	50.0	52	42	80.7
12	Tachihara et al [16]	2017	6	4	66.7	12	11	91.7
13	Nakai et al [23]	2017	48*^5^	27	56.3	26*^5^	20	76.9
14	Nakai et al [24]	2017	130*^5^	85	65.4	124*^5^	82	66.1
15	Ito et al [32]	2021	7*^6^	5	71.4	12*^6^	7	58.3
16	Ito et al [27]	2022	89*^7^	52	58.4	273*^7^	221	80.9
17	Oki et al [34]	2022	159	79*^8^	49.7	141	87*^8^	61.7

Discussion

A recent meta-analysis demonstrated that the use of novel techniques such as EBUS-GS is associated with improved diagnostic yield of bronchoscopy (up to 70%) [35]. Conversely, a study from Norway demonstrated a low detection rate for cancer in PPLs, although bronchoscopy was performed by bronchoscopists with varying levels of expertise [3]. Although EBUS is increasingly being used to guide sample collection in several countries, guide sheaths are not usually used in countries other than Japan. In Japan, guide sheaths are widely used during radial probe EBUS-guided transbronchial biopsy of PPLs [36]. Oki et al. demonstrated that the use of a guide sheath enhanced the diagnostic yield for small PPLs [34]. Himeji et al. observed that EBUS-GS was useful for detecting pulmonary Actinomyces graevenitzii infection and invasive mucinous adenocarcinoma [37,38]. To the best of our knowledge, this review is the first to evaluate the usefulness of EBUS-GS when bronchoscopy is performed by bronchoscopists in Japan with varying levels of expertise. The limitation of this research is that it included the analytical methods and heterogeneity among individual studies.

Recent studies of the use of EBUS-GS for PPLs have reported diagnostic yields ranging from as low as 40% to as high as 90%. Surprisingly, the diagnostic yield was not significantly different for guided bronchoscopy procedures performed before and after 2012 [39]. Robotic bronchoscopy was approved by the FDA in 2018 and has received significant attention [40]. The combined use of robotic bronchoscopy and other technologies, such as cone-beam computed tomography (CT), prevents CT-to-body divergence to optimize biopsy tool-in-lesion [41]. In the present review, the diagnostic yield for 2996 malignant lesions, including lung cancer, was 73.6% (range: 61.9%-88.2%) when bronchoscopy was performed by Japanese operators. In Japan, bronchoscopy is typically performed under moderate sedation with opioids and/or benzodiazepines [42]. However, in several other countries, it is performed under general anesthesia [43]. In addition, Japanese bronchoscopists are experienced in performing procedures for more peripheral and smaller nodules compared to bronchoscopists from other countries. The Japanese technique of bronchoscopy is high quality, because of the corresponding medical education strategies. The society operates independently from other respiratory societies, establishes an accredited specialist training system, and provides an annual program for continuous education to specialists. This unique approach is notably distinct from other countries. Moreover, a multimodal approach combining EBUS-GS, ultrathin bronchoscopy, and virtual bronchoscopic navigation can improve the diagnosis of PPLs. A 3.0 mm ultrathin bronchoscope has recently been used in clinical practice in Japan [10]. Moreover, in clinical trials, virtual bronchoscopic navigation is associated with a higher diagnostic yield than nonvirtual bronchoscopic navigation [28,35]. In the present review, the diagnostic yield of EBUS-GS was 65.4% (range: 56.7-77.1%) for 752 ground-glass nodules. Such nodules are typically evaluated using EBUS-GS (GuideSheath Kit 2, K403; Olympus) and large transbronchial biopsy forceps (Radial Jaw™4P; Boston Scientific) [44]. For targeted therapies of lung cancer patients based on next-generation sequencing (NGS), sufficiently large tissue specimens are required during bronchoscopy. In Japan, NGS is typically performed using the Oncomine Dx Target Test and AmoyDx® Pan Lung Cancer PCR Panel. The use of the lung cancer compact panel, approved by the Japanese Pharmaceutical Affairs in November 2022 as the third multi-gene panel test, is associated with a high success rate for genetic analysis [45]. During EBUS-GS, small forceps are typically used, leading to insufficient sample collection for NGS. The combination of EBUS-GS and the lung cancer compact panel is a promising diagnostic strategy for lung cancer [46].

## Conclusions

In Japan, EBUS-GS is effective for the diagnosis of malignant and benign PPLs. The Japanese technique of bronchoscopy is of high quality because of the corresponding medical education strategies. A multimodality approach (such as ultrathin bronchoscopy, virtual bronchoscopic navigation, cryobiopsy, and robotic bronchoscopy) is needed to improve its performance.

## References

[1] Kurimoto N, Miyazawa T, Okimasa S, Maeda A, Oiwa H, Miyazu Y, Murayama M (2004). Endobronchial ultrasonography using a guide sheath increases the ability to diagnose peripheral pulmonary lesions endoscopically. Chest.

[2] Huang CT, Ho CC, Tsai YJ, Yu CJ, Yang PC (2009). Factors influencing visibility and diagnostic yield of transbronchial biopsy using endobronchial ultrasound in peripheral pulmonary lesions. Respirology.

[3] Roth K, Eagan TM, Andreassen AH, Leh F, Hardie JA (2011). A randomised trial of endobronchial ultrasound guided sampling in peripheral lung lesions. Lung Cancer.

[4] Kikuchi E, Yamazaki K, Sukoh N (2004). Endobronchial ultrasonography with guide-sheath for peripheral pulmonary lesions. Eur Respir J.

[5] Tamiya M, Sasada S, Kobayashi M (2011). Diagnostic factors of standard bronchoscopy for small (≤15 mm) peripheral pulmonary lesions: a multivariate analysis. Intern Med.

[6] Oki M, Saka H, Kitagawa C, Kogure Y, Murata N, Adachi T, Ando M (2012). Randomized study of endobronchial ultrasound-guided transbronchial biopsy: thin bronchoscopic method versus guide sheath method. J Thorac Oncol.

[7] Minami D, Takigawa N, Morichika D (2015). Endobronchial ultrasound-guided transbronchial biopsy with or without a guide sheath for diagnosis of lung cancer. Respir Investig.

[8] Minezawa T, Okamura T, Yatsuya H (2015). Bronchus sign on thin-section computed tomography is a powerful predictive factor for successful transbronchial biopsy using endobronchial ultrasound with a guide sheath for small peripheral lung lesions: a retrospective observational study. BMC Med Imaging.

[9] Hayama M, Izumo T, Matsumoto Y, Chavez C, Tsuchida T, Sasada S (2015). Complications with endobronchial ultrasound with a guide sheath for the diagnosis of peripheral pulmonary lesions. Respiration.

[10] Oki M, Saka H, Ando M (2015). Ultrathin bronchoscopy with multimodal devices for peripheral pulmonary lesions. A randomized trial. Am J Respir Crit Care Med.

[11] Chavez C, Sasada S, Izumo T, Watanabe J, Katsurada M, Matsumoto Y, Tsuchida T (2015). Endobronchial ultrasound with a guide sheath for small malignant pulmonary nodules: a retrospective comparison between central and peripheral locations. J Thorac Dis.

[12] Okachi S, Imai N, Imaizumi K (2016). Factors affecting the diagnostic yield of transbronchial biopsy using endobronchial ultrasonography with a guide sheath in peripheral lung cancer. Intern Med.

[13] Izumo T, Matsumoto Y, Chavez C, Tsuchida T (2016). Re-biopsy by endobronchial ultrasound procedures for mutation analysis of non-small cell lung cancer after EGFR tyrosine kinase inhibitor treatment. BMC Pulm Med.

[14] Uchimura K, Yamasaki K, Ishimoto H (2016). [Factors associated with diagnostic yield of endobronchial ultrasonography with a guide sheath for peripheral lung cancer]. J UOEH.

[15] Asano F, Ishida T, Shinagawa N (2017). Virtual bronchoscopic navigation without X-ray fluoroscopy to diagnose peripheral pulmonary lesions: a randomized trial. BMC Pulm Med.

[16] Tachihara M, Tamura D, Kiriu T (2017). Bronchoscopy using virtual navigation and endobronchial ultrasonography with a guide sheath (EBUS-GS) with or without fluoroscopy for peripheral pulmonary lesions. Kobe J Med Sci.

[17] Kajikawa S, Imai N, Okachi S (2019). Diagnostic contribution of cytological examination to endobronchial ultrasound-guided transbronchial biopsy for lung malignancies. Nagoya J Med Sci.

[18] Ito T, Okachi S, Iwano S, Kinoshita F, Wakahara K, Hashimoto N, Chen-Yoshikawa TF (2022). Diagnostic value and safety of endobronchial ultrasonography with a guide sheath transbronchial biopsy for diagnosing peripheral pulmonary lesions in patients with interstitial lung disease. J Thorac Dis.

[19] Kurihara Y, Tashiro H, Takahashi K (2022). Factors related to the diagnosis of lung cancer by transbronchial biopsy with endobronchial ultrasonography and a guide sheath. Thorac Cancer.

[20] Izumo T, Sasada S, Chavez C, Tsuchida T (2013). The diagnostic utility of endobronchial ultrasonography with a guide sheath and tomosynthesis images for ground glass opacity pulmonary lesions. J Thorac Dis.

[21] Ikezawa Y, Sukoh N, Shinagawa N, Nakano K, Oizumi S, Nishimura M (2014). Endobronchial ultrasonography with a guide sheath for pure or mixed ground-glass opacity lesions. Respiration.

[22] Izumo T, Sasada S, Chavez C, Matsumoto Y, Tsuchida T (2015). Radial endobronchial ultrasound images for ground-glass opacity pulmonary lesions. Eur Respir J.

[23] Nakai T, Izumo T, Matsumoto Y, Tsuchida T (2017). Virtual fluoroscopy during transbronchial biopsy for locating ground-glass nodules not visible on X-ray fluoroscopy. J Thorac Dis.

[24] Nakai T, Matsumoto Y, Suzuk F, Tsuchida T, Izumo T (2017). Predictive factors for a successful diagnostic bronchoscopy of ground-glass nodules. Ann Thorac Med.

[25] Ikezawa Y, Shinagawa N, Sukoh N (2017). Usefulness of endobronchial ultrasonography with a guide sheath and virtual bronchoscopic navigation for ground-glass opacity lesions. Ann Thorac Surg.

[26] Shinagawa N, Nakano K, Asahina H (2012). Endobronchial ultrasonography with a guide sheath in the diagnosis of benign peripheral diseases. Ann Thorac Surg.

[27] Nakashima K, Misawa M, Otsuki A, Narita K, Otsuka Y, Matsue K, Aoshima M (2022). Efficacy and safety of endobronchial ultrasonography with a guide-sheath for acute pulmonary lesions in patients with haematological diseases. Intern Med.

[28] Ishida T, Asano F, Yamazaki K (2011). Virtual bronchoscopic navigation combined with endobronchial ultrasound to diagnose small peripheral pulmonary lesions: a randomised trial. Thorax.

[29] Ishida M, Suzuki M, Furumoto A, Tsuchihashi Y, Ariyoshi K, Morimoto K (2012). Transbronchial biopsy using endobronchial ultrasonography with a guide sheath increased the diagnostic yield of peripheral pulmonary lesions. Intern Med.

[30] Sakamoto T, Kodani M, Takata M (2015). A novel point-of-care system for high-speed real-time polymerase chain reaction testing for epidermal growth factor receptor mutations in bronchial lavage fluids after transbronchial biopsy in patients with non-small cell lung cancer. Int J Oncol.

[31] Hayama M, Okamoto N, Suzuki H (2016). Radial endobronchial ultrasound with a guide sheath for diagnosis of peripheral cavitary lung lesions: a retrospective study. BMC Pulm Med.

[32] Ito T, Kimura T, Kataoka K, Okachi S, Wakahara K, Hashimoto N, Kondoh Y (2021). A pilot study of transbronchial biopsy using endobronchial ultrasonography with a guide sheath in the diagnosis of peripheral pulmonary lesions in patients with interstitial lung disease. Diagnostics (Basel).

[33] Ito T, Okachi S, Kimura T (2021). Endobronchial ultrasonography with a guide sheath transbronchial biopsy for diagnosing peripheral pulmonary lesions within or near fibrotic lesions in patients with interstitial lung disease. Cancers (Basel).

[34] Oki M, Saka H, Imabayashi T (2022). Guide sheath versus non-guide sheath method for endobronchial ultrasound-guided biopsy of peripheral pulmonary lesions: a multicentre randomised trial. Eur Respir J.

[35] Wang Memoli JS, Nietert PJ, Silvestri GA (2012). Meta-analysis of guided bronchoscopy for the evaluation of the pulmonary nodule. Chest.

[36] Takashima Y, Oki M (2023). Endobronchial ultrasound with a guide sheath during bronchoscopy for peripheral pulmonary lesions. Expert Rev Respir Med.

[37] Himeji D, Hara S, Kawaguchi T, Tanaka GI (2018). Pulmonary Actinomyces graevenitzii infection diagnosed by bronchoscopy using endobronchial ultrasonography with a guide sheath. Intern Med.

[38] Kawaguchi T, Himeji D, Beppu K, Marutsuka K (2021). Snowball-like appearance on radial endobronchial ultrasonography in a patient with invasive mucinous adenocarcinoma. Respirol Case Rep.

[39] Nadig TR, Thomas N, Nietert PJ (2023). Guided bronchoscopy for the evaluation of pulmonary lesions: an updated meta-analysis. Chest.

[40] Diddams MJ, Lee HJ (2023). Robotic bronchoscopy: review of three systems. Life (Basel).

[41] Duke JD, Reisenauer J (2023). Robotic bronchoscopy: potential in diagnosing and treating lung cancer. Expert Rev Respir Med.

[42] Minami D, Takigawa N (2023). Safe sedation during diagnostic and therapeutic flexible bronchoscopy in Japan: a review of the literature. Respir Investig.

[43] Skinner TR, Churton J, Edwards TP (2021). A randomised study of comfort during bronchoscopy comparing conscious sedation and anaesthetist-controlled general anaesthesia, including the utility of bispectral index monitoring. ERJ Open Res.

[44] Kunimasa K, Tachihara M, Tamura D (2016). Diagnostic utility of additional conventional techniques after endobronchial ultrasonography guidance during transbronchial biopsy. Respirology.

[45] Bernardini A, Dueñas M, Martín-Soberon MC (2022). Genomic landscape of vinflunine response in metastatic urothelial cancer. Cancers (Basel).

[46] Kunisama K, Tamiya M, Inoue T (2024189). Clinical application of the lung cancer compact PanelTM using various types of cytological specimens in patients with lung cancer. Lung Cancer.

